# Minimally invasive treatment of both-column acetabular fractures through the Stoppa combined with iliac fossa approach

**DOI:** 10.1038/s41598-017-08724-1

**Published:** 2017-08-14

**Authors:** Ruipeng Zhang, Yingchao Yin, Shilun Li, Zhiyong Hou, Juan Wang, Wei Chen, Yingze Zhang

**Affiliations:** Department of Orthopaedic Surgery, Third Hospital of Hebei Medical University, Ziqiang Road 139, Shijiazhuang, 050051 Hebei P.R. China

## Abstract

Both-column fractures are the most complicated group of acetabular fractures. Although great progress of surgical technique has been made, the choice of approach is controversial. All the fragments could be exposed and managed through combined ilioinguinal and Kocher-Langenbeck (IL+KL) approaches, which has been widely used to conduct the both-column fractures. However, the clinical popularization may be restricted for high rate of complication. Most internal area of the hemipelvis could be exposed through Stoppa combined with iliac fossa (S+IF) approach. The majority of both-column fractures were managed through IL+KL approaches or S+IF approach in our institution. The comparison of the two surgical methods was done in this study. The purpose is to examine whether S+IF approach could achieve the satisfactory reduction and fixation for both-column fractures.

## Introduction

Both-column fractures are the most complicated of all acetabular fractures because the entire weight-bearing articular surface is detached from the sacroiliac joint, and the fracture lines involve multiple planes^1–3^. Anatomical reduction and firm internal fixation are the main actions required to achieve a good outcome for acetabular fractures^3–5^. Although great progress has been achieved for both-column fractures, the choice of surgical approach is still controversial^6^. Some experts believed that ilioinguinal (IL) and Kocher-Langenbeck (KL) approaches were indispensable because neither the IL nor KL approach alone was capable of exposing and managing all of the fragments^2, 3, 5, 7–9^. Orthopedists, however, were confronted with some thorny problems with the approaches, including a longer surgical time, larger surgical incision, and intraoperative repositioning of the patient^5, 10^.

The entire iliac wing could be exposed through iliac fossa (IF) approach (lateral window of IL approach). The area from the sacroiliac joint to the pubic symphysis could be exposed using the Stoppa approach, which has been widely used to treat pelvic and acetabular fractures^4, 11^. However, there is little literature that describes the treatment of both-column fractures via the Stoppa combined with iliac fossa (S+IF) approach. Therefore, we compared the clinical outcomes of two surgical methods: IL+KL approaches and S+IF approach. The purpose is to examine whether the minimally invasive (S+IF) approach could achieve satisfactory reduction and fixation for both-column fractures.

## Results

### Surgical comparison

Average surgical time was 84.17 minutes (60~120 minutes) in Group A and 116.09 minutes (90~150 minutes) in Group B (*P* = 0.000). Average blood loss was determined at a mean 466.67 ml (200~1000 ml) in Group A and at a mean 656.25 ml (200~1600 ml) in Group B (P = 0.002) (Table 1). There was no poor or secondary congruence for the reduction in this study. 25 cases obtained anatomic reduction and 11 cases got imperfect reduction in Group A, 24 cases obtained anatomic reduction and 8 cases got imperfect reduction in Group B (P > 0.05).

**Table 1:** Comparison of some indexes in two groups was presented.

	Group A	Group B	*P* values
Surgery time (min)	84.17 ± 16.37	116.09 ± 16.00	*P* = 0.000
Blood loss (ml)	466.67 ± 169.03	656.25 ± 285.04	*P* = 0.002
HHS	84.03 (69~97)	85.69 (72~98)	*P* = 0.365
Merle D’Aubigné	15.25 (13~18)	15.56 (13~18)	*P* = 0.324
Hip Flexion	95.28 ± 16.03	97.50 ± 16.56	*P* = 0.576
Hip Extension	9.70 ± 2.29	10.16 ± 2.52	*P* = 0.431
Complication rate	4/36	10/32	*P* = 0.04

### Functional comparison

At the final follow-up, the mean hip extension was 9.69° ± 2.29° in Group A, while the value was 10.16° ± 2.52° in Group B (*P* = 0.431). The mean hip flexion was 95.28° ± 16.03° in Group A and the value was 97.50° ± 16.56° in Group B (*P* = 0.576). The mean HHS was 84.03 ± 7.28 in group A and the value was 85.69 ± 7.73 in Group B (*P* = 0.365). The mean Merle D’Aubigné score was 15.25 ± 1.34 in Group A, while, the value was 15.56 ± 1.24 in group B (*P* = 0.324) (Table 1).

### Comparison of complication

The complication rates (injury of vessels or nerves, postoperative infection) were 11.11% in group A (4 patients) and 31.25% in group B (10 patients) (*P* = 0.04) (Table 1). In group A, lateral femoral cutaneous nerve (LFCN) injury existed in two cases postoperatively. The resulting paralysis of the lateral thigh disappeared after 3 months of conservative treatment. The obturator nerve was damaged in two patients intraoperatively but had recovered 3 months later. In group B, LFCN injury developed in three patients, the symptoms of which disappeared after 3 months of conservative treatment. Sciatica symptoms were experienced by one patient but had disappeared 11 months later. Femoral nerve injury developed in two patients, and one patient’s symptoms were still apparent at the final follow-up. Intraoperative injury of the femoral vein occurred in one patient. Haematomas developed in two patients, with the symptoms disappearing after treating the puncture. Soft tissue infection developed in one patient and the purulence was eliminated after debridement. Non-union was not seen in this study (Table 2).

**Table 2:** Postoperative complications of the two groups were presented.

	Group A	Group B
Lateral femoral cutaneous nerve injury	2	3
Sciatic nerve problem	—	1
Obturator nerve injury	2	—
Femoral nerve injury	—	2
Femoral vessel injury	—	1
Haematoma	—	2
Soft tissue infection	—	1

## Discussion

Our results showed that effective reduction of the both-column acetabular fractures with displaced fragments was obtained through S+IF approach. In addition, the incision was smaller and the rate of intraoperative complications was lower than occurred with the IL+KL approaches.

**Figure 1:**
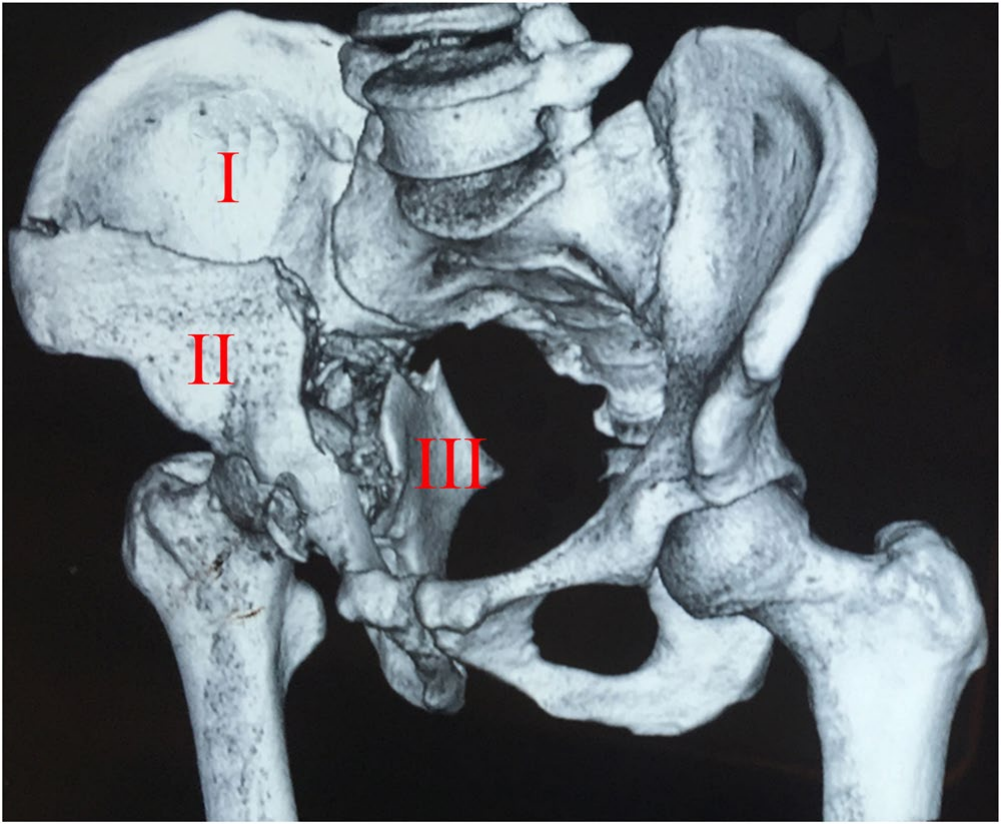
The hemipelvis was divided into three main fragments. Fixed posterior iliac fragment (I), anteromedially displaced iliopubic fragment (II), medially displaced ischial fragment (III).

Both-column fractures, characterized by a “floating acetabulum”, present the most complicated pattern of all acetabular fractures^2, 12^. The “T” or “Y” shaped fractures of the hemipelvis formed by two main converging lines are composed of three main fragments: fixed posterior iliac fragment, anteromedially displaced iliopubic fragment and medially displaced ischiadic fragment (Fig. 1)^7, 13^. The main fragments may be further crushed and divided for the patients who have suffered serious trauma.

**Figure 2:**
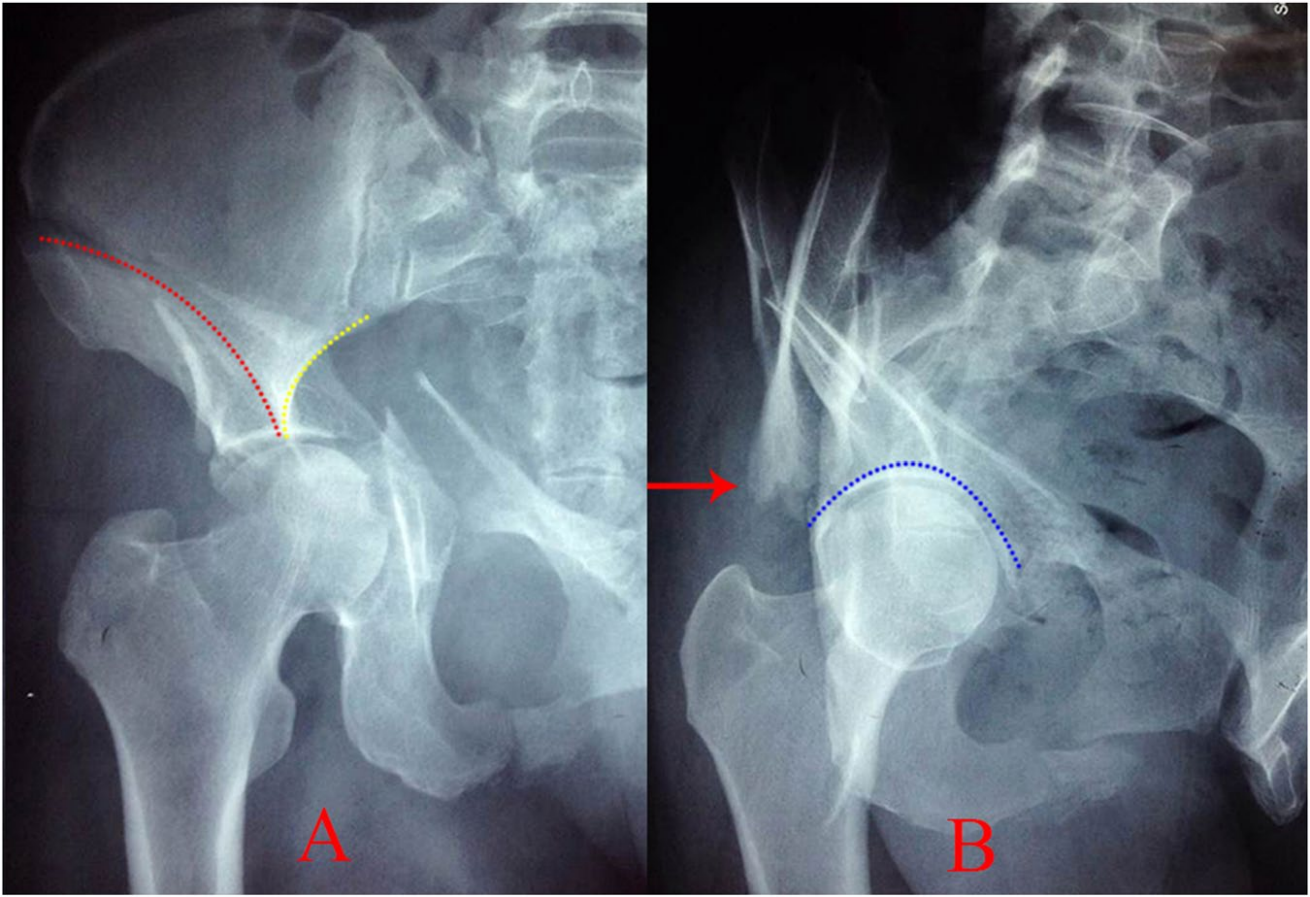
The radiographic characteristics of both-column fractures. (**A**) Iliac wing fracture line (red dotted line) and curved appearance (yellow dotted line), (**B**) the false congruence (blue dotted line) and the spur sign (arrow) are apparent.

The iliac wing fracture, curved appearance, false congruence and spur sign are radiographic characteristics of both-column acetabular fractures (Fig. 2). The hemipelvis including the entire iliac wing and the area from the sacroiliac joint to the pubic symphysis could be adequately exposed through S+IF approach^8, 11, 14–17^. The reduction and fixation between the fixed posterior iliac fragment and the anteromedially displaced iliopubic fragment could be performed using the iliac fossa approach.

**Figure 3:**
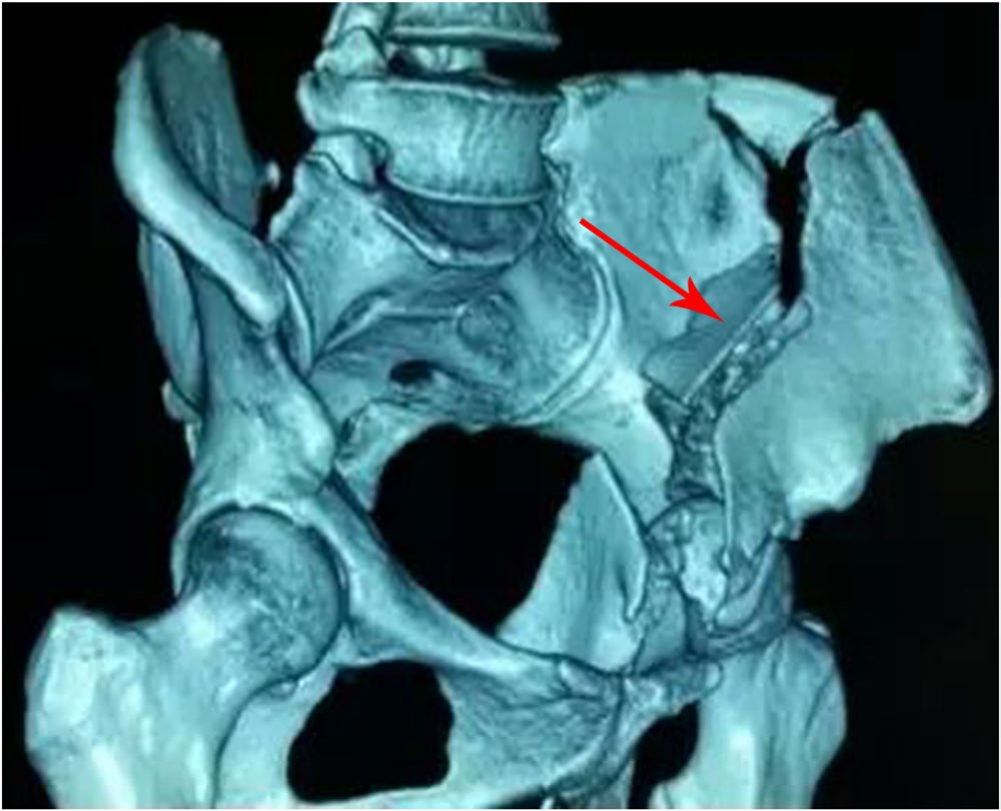
Minor fragment called the “key stone” was indicated by red arrow.

**Figure 4:**
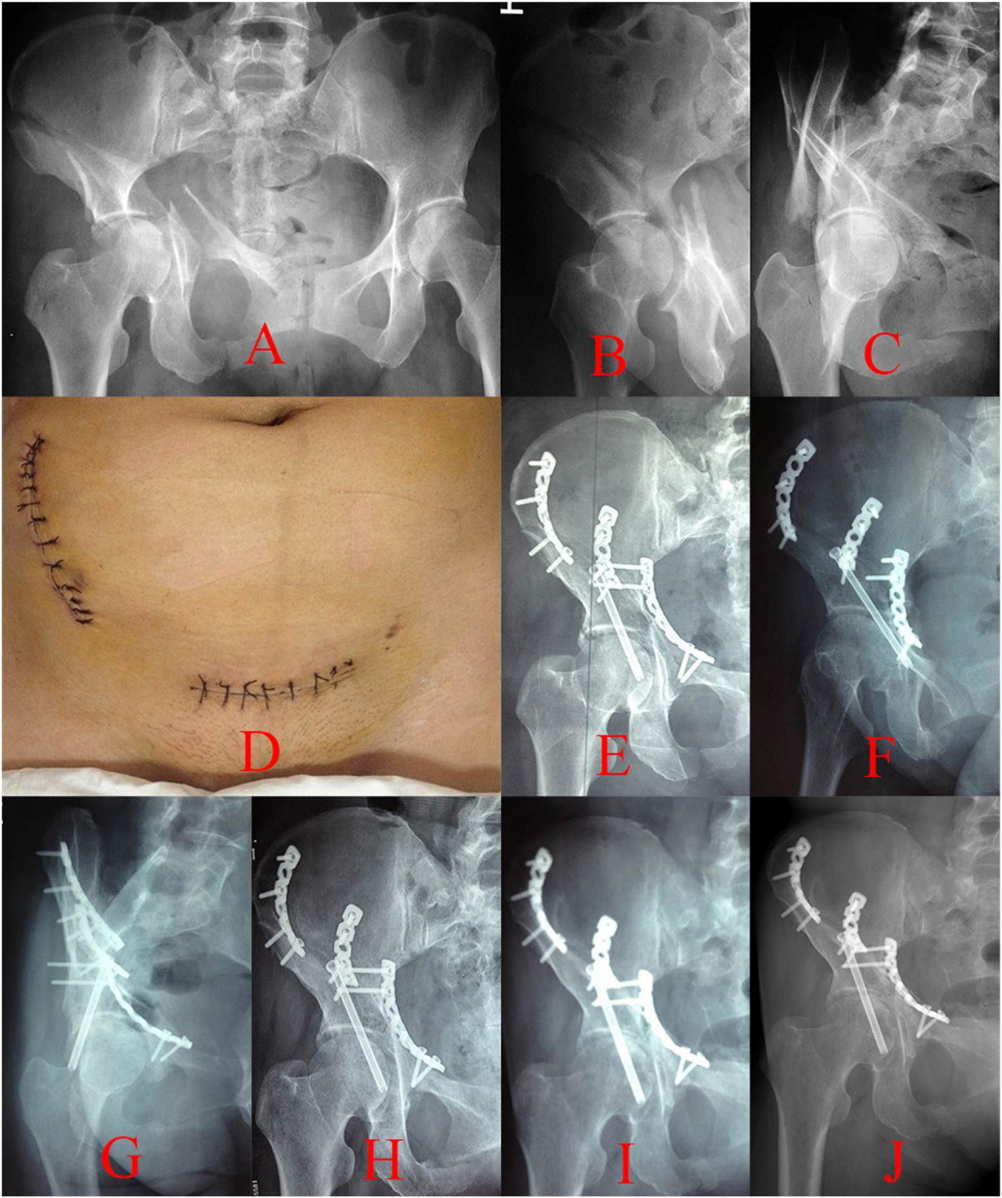
A patient treated with the S+IF approach. (**A**–**C**) Preoperative radiographs of pelvis, (**D**) surgical incision of the S+IF approach, (**E**) postoperative anteroposterior projection, (**F**) iliac oblique projection, (**G**) obturator oblique projection. (**H**–**J**) anteroposterior projections in the first, third and sixth months’ follow-up.

**Figure 5:**
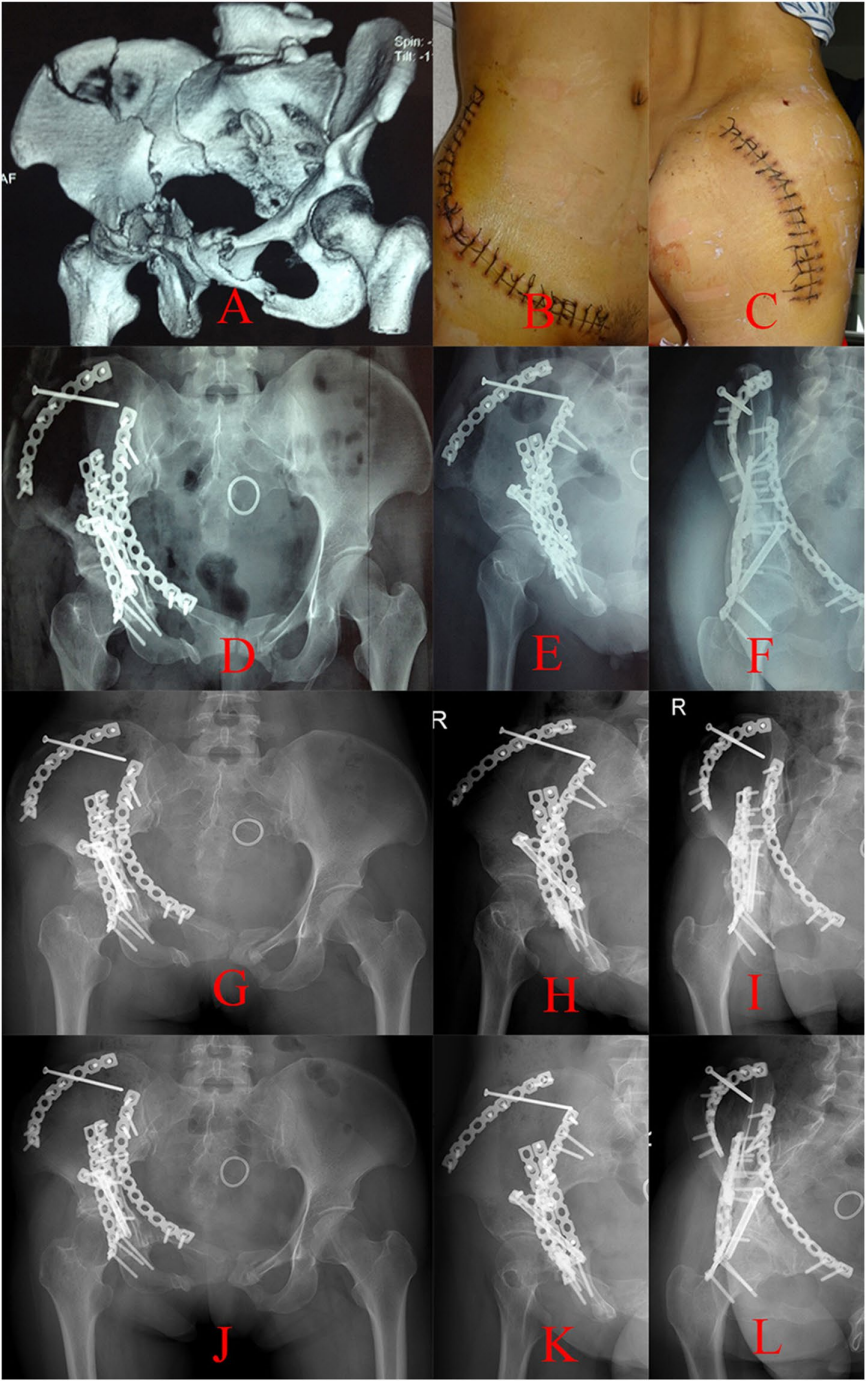
A patient treated with combined IL+KL approaches. (**A**) Preoperative three-dimensional reconstructions, (**B**,**C**) surgical incision of IL+KL approaches, (**D**–**F**) anteroposterior, iliac oblique and obturator oblique projections in the first month’s follow-up, (**G**–**I**) anteroposterior, iliac oblique and obturator oblique projections in the third month’s follow-up, (**J**–**L**) anteroposterior, iliac oblique and obturator oblique projections in the sixth month’s follow-up.

A minor fragment called the “key stone” is always present in the iliac fossa. Its reduction is the pivotal step of this surgery (Fig. 3). The anteromedially displaced iliopubic fragment and medially displaced ischial fragment cannot be reduced unless the reduction of “key stone” was achieved. The quadrilateral plate forms the medial side of the acetabulum and could be reduced via the Stoppa approach from the contralateral side with traction of the lower limbs. The intersection of the main fracture lines, located in the greater sciatic notch, could be exposed via the Stoppa approach. The greater sciatic notch is regarded as a reference during fragment reduction, and the plate is placed at the pelvic brim^4, 11^. In addition, an anterograde screw is an alternative method for fixing anterior and posterior fragments when two columns are involved^18^ (Figs 4 and 5).

IL+KL approaches are classic surgical procedures, which could be used to reduce displaced fragments of both columns simultaneously with the lateral decubitus. This combination, however, is accompanied by a high rate of iatrogenic complications, such as vessel and nerve injuries^2, 19^. Furthermore, intraoperative manipulation of fragments may not be easy because the lateral decubitus is not ideal for either of the two approaches^2, 5^.

Moroni *et al*. performed staged surgery using the IL+KL approaches to obtain better exposure of the fragments. After evaluating the fracture pattern by radiography, surgery was conducted first at the more displaced and comminuted column^16^. Then, the patient was turned over to manage the other column^2, 16^. With this technique, however, the reduction of the opposite column might be impeded for the fixation of fragments. Also, as the patient was repositioned, the intraoperative adjustment of screws may become difficult, which may limit its clinical use as no one wants to face this dilemma. The S+IF approach could be performed with the supine position during the operation. Thus, intraoperative repositioning and a second round of disinfection are avoided, which would greatly decrease the surgical time and blood loss.

Gänsslen and Krettek reported that good outcomes could be obtained using the ilioinguinal approach alone^20^. When using the standard ilioinguinal approach, however, the femoral neurovascular bundle might be injured during the dissection of the second window^17, 21^. In addition, it was difficult to reduce the posterior fragments because direct visualization of the quadrilateral plate was impossible. However, the displaced quadrilateral plate could be visualized and reduced with palpation via the Stoppa approach^22^. Thus, ilioinguinal approach might result in more iatrogenic injury and poorer reduction of quadrilateral plate than the S+IF approach.

The displaced fragments of both-column acetabular fractures are mainly caused by an impact between the acetabulum and the femoral head. Reduction of the acetabulum is difficult to complete until after the medially displaced femoral head is reduced, which is easily achieved with the S+IF approach. Thus, satisfactory outcomes could be obtained while keeping the surgical time, blood loss, and complication rate low.

During managing the displaced acetabular fragments, iatrogenic injury of the LFCN may be accompanied because of its highly variable course and branches^23, 24^. Relative symptoms of LFCN irritation such as paralysis and pain at anterolateral thigh could be detected postoperatively^25^. Fang *et al*. reported that explicit identification of LFCN could not reduce the incidence of its intraoperative injury^25^. Our experience showed that protection of LFCN may be obtained if the separation of soft tissues was conducted along the iliac periosteum. In addition, LFCN injury was regarded as a kind of self-limiting complication and the symptoms could disappear after months of conservative treatment^25^.

As a kind of serious fractures, both-column fractures are always caused by high-energy trauma. Thus, polytrauma including other organs injury and lower extremity fractures may be accompanied, which would affect the therapeutic strategies. Cardio-pulmonary resuscitation is the overriding procedure. Use of pelvic pocket timely is advocated to decrease the bleeding of fracture area. Then, injury involving important organs such as brain and gastrointestinal should be managed. Skeletal traction is ought to be done after the admission of hospital to lower the difficulty of intraoperative reduction. Fracture treatment is conducted after the stable vital signs have been obtained. Fractures involving femur and tibia should be processed prior to pelvis, which would facilitate intraoperative traction for the reduction of acetabular fracture.

As high-energy trauma, the both-column acetabular fractures may be accompanied by posterior pelvic ring fracture, which means the whole pelvic ring was injured. The disruption of pelvic ring should be managed prior to the both-column acetabular fractures if the instability of posterior ring was accompanied. Some fixation methods including lumbopelvic rod, reconstruction plate and sacroiliac screw may be applied to stabilize the posterior pelvic ring^26, 27^. For the patients with serious vertical detachment of posterior ring, open reduction and lumbopelvic fixation are essential for good prognosis. Posterior reconstruction plate or percutaneous sacroiliac screw may be inserted for the sacral fractures without vertical instability. The dislocation of sacroiliac joint or iliac fracture could be directly visualized and reduced through iliac fossa approach, sacroiliac screw or anterior plate could be placed. However, the anterior pelvic ring injury including pubis symphysis disruption or bilateral pubic rami fractures could be managed through the Stoppa approach. Both-column acetabular fractures could be processed through S+IF approach after the management of pelvic injury has been accomplished.

The defects of S+IF approach may involve inadequate exposure of the entire posterior column and the risk of obturator neurovascular bundle or urocystic injury^11^. For the patients with seriously displaced or comminuted posterior fracture, it was indispensable to perform additional KL approach to manage the fragments. Urine with multiple bacteria would flow into soft tissue space after the rupture of bladder, which may lead to disastrous infection for the internal fixation. The plate was always placed at the inner surface of pelvic ring through the Stoppa approach, then, the surrounding tissues including corona mortis and obturator neurovascular bundle may be damaged during the surgical procedure.

There are some deficiencies in this study. As a retrospective study, the patients of are not randomly divided into two groups. The study has relative small sample size, which may not represent the characters of all both-column fractures. It has been reported that single IL or KL approach could manage the both-column fractures in the past. However, relative control groups were not included in this study. We would recruited more patients to assess the feasibility of (S+IF) approach for both-column fractures.

In summary, as a minimally invasive alternative, S+IF approach is recommended to manage the both-column fractures.

## Methods

### Ethical statements

Informed consent was obtained from all the patients of the study. We declared that all work including surgical methods and review were performed in accordance with the guidelines of the institutional review board of Third Hospital of Hebei Medical University. The experimental protocols were approved by institutional review board of Third Hospital of Hebei Medical University and the registered number was 2014-006-1.

### Grouping of the patients

We conducted a retrospective study that explored an alternative surgical approach for both-column acetabular fractures. We reviewed the medical records of 68 patients with both-column acetabular fractures who had undergone S+IF or IL+KL surgical method during the period from January 2009 to January 2014. Patients with a preoperative range of motion (ROM) deficiency of the hip, open fractures, or had not completed 1 year of follow-up were excluded from the study. Overall, 68 patients met the criteria for inclusion. They were divided into two groups according to the surgical approaches used. In all, 36 patients treated with the S+IF approach (group A) with a mean follow-up of 22.42 months (Fig. 4) and 32 patients treated with the IL+KL approaches (group B) with a mean follow-up of 24.16 months (Fig. 5) were reviewed in the study.

### Surgical techniques of two groups

Supine position was preferred for the patients received S+IF approach. The incision of iliac fossa approach began at the middle of the iliac crest to anterior superior iliac spine. The abdominal muscles were separated and retracted medially. Direct access to inner surface of iliac wing from the sacroiliac joint to the anterior superior iliac spine could be obtained through iliac fossa approach. Fragments of iliac wing should be managed firstly because its integrity served as reference for the reduction of other fragments. Iliopubic fragment could be reduced with a continuing traction force of lower extremity. The Stoppa incision was performed in the region 1–2 cm superior to pubic symphysis. Then, longitudinal split of linea alba (the junction of bilateral rectus fibers) was conducted. Standing at contralateral side, pelvic ring and quadrilateral surface could be exposed through Stoppa approach. Ischiadic fragment could be reduced with an outward force from a ball-spiked pusher through Stoppa approach. An infrapectineal plate could be placed to stabilize the fragments. Anterograde lag screws may be inserted to fix the anterior and posterior columns with the guidance of the C-arm radiograph. Suture of surgical incision could be performed after the internal fixation was accomplished.

Some tips were proposed to lower the incidence of iatrogenic injury. Separation of soft tissues along the iliac periosteum was recommended to protect LFCN. Ligation of corona mortis should be done to avoid intraoperative uncontrolled bleeding. Flexed position of hip joint was maintained during traction to reduce the tension of femoral vessels.

Lateral decubitus position was preferred for IL+KL approaches. The column more displaced or comminuted was managed preferentially. Posterior and anterior fragments were disposed through KL and IL approach respectively in group B.

The KL approach incision began 4 cm anteriorly to the posterior superior iliac spine and ran to the great trochanter with an externally convex curve. Soft tissues including gluteus maximus and extorsion muscles were separated and protected. Two Hoffman retractors were placed in two ischiatic incisures to protect the sciatic nerve. Then, the entire posterior column and wall could be exposed and palpated. The greater ischial notch could serve as a reduction mark of posterior column fragments. The detachment of posterior wall was ought to be managed after the stabilization of posterior column. The screws should be inserted distantly to acetabulum in case of the penetration into hip joint.

The incision of IL approach begins at the midpoint of iliac crest and is continued anteriorly and distally from ASIS to the pubic tubercle. Lacuna vasorum (femoral vessels) and lacuna musculorum (lateral femoral cutaneous nerve, psoas tendon, and femoral nerve) divided the IL approach into three windows. The region could be exposed through the lateral window of IL approach was similar to iliac fossa approach. The middle window is able to provide visualization of quadrilateral surface and pelvic brim in profile. Ipsilateral pubic ramus could be almost entirely exposed through the medial window of IL approach. The anterior fragments could be managed by retracting lacuna vasorum and lacuna musculorum through IL approach. Not only infrapectineal but also suprapectineal plate could be placed through IL approach. The incision would be sutured after the reduction and fixation of fragments were accomplished.

Sciatic nerve superior gluteal artery should be protected when reduced the posterior fragments. Opening inguinal canal and handling the contents of femoral triangle are required, which may be technique demanding for most surgeons.

### Comparison of two groups

The demographic data of the two groups is shown in Table 3. All of the surgical procedures were conducted by the same team in this study. The patients’ charts were surveyed for the surgical time, blood loss, and quality of the reduction. The reduction of the articular surface was graded based on the immediately postoperative radiographs as anatomic (less than 1 mm of displacement), imperfect (2–3 mm of displacement), poor (greater than 3 mm of displacement), or secondary congruence as described by Matta^28^. Identical therapeutic protocols were performed for the two groups. Anteroposterior, obturator oblique, and iliac oblique radiographs of the pelvis were conducted to evaluate the status of the fracture union. Follow-up was performed at one, two, three months postoperatively and every three months thereafter. Daily exercise was guided by their individual status. ROM, Harris Hip Score (HHS), and Merle D’Aubigné score were recorded at the final review. Complications such as infection, neurovascular injuries, and haematoma were also recorded.

**Table 3:** Demographic data of two groups.

	Group A	Group B	*P* values
Age	42.06 years	44.06 years	0.415
Gender F/M	10/26	12/20	0.392
Time to surgery	7.25 days	6.41 days	0.219
Mean follow-up	22.42 months	24.16 months	0.107

### Statistical analysis

The results are presented as the mean ± standard deviation. The complication rates of two groups were determined by Chi-square test. Other differences between the two groups were determined by t-tests. A value of *P* ≤ 0.05 was considered to indicate significance. The data described above was processed by SPSS 13.0 software (SPSS Chicago, IL, USA).
